# Decrypting network physiology for clinical practice

**DOI:** 10.3389/fnetp.2026.1854895

**Published:** 2026-06-26

**Authors:** Reece Munson, Megan Sands, Sarvesh Srinivasan, Christos Charalambous, Amar Singh Bhogal

**Affiliations:** 1 St Helier Hospital, Carshalton, United Kingdom; 2 Lister Hospital, London, United Kingdom; 3 Whittington Hospital, London, United Kingdom; 4 Royal Free London NHS Foundation Trust, London, United Kingdom; 5 University College London, London, United Kingdom; 6 Frimley Health NHS Foundation Trust, Frimley, United Kingdom

**Keywords:** COPD, chronic obstructive pulmonary disease, liver cirrhosis, network physiology, OSA (obstructive sleep apnea), oxygen saturation variability, sepsis, transfer entropy

## Abstract

Network physiology is a growing and expanding field analysing the exchange of information across body systems. The main goal is to understand and represent the body in a complex network with the hopes that these tools can bring real clinical value. These concepts may seem foreign to most clinicians, but we intuitively understand this in day-to-day practice. The hypoxic patient with asthma presenting with tachycardia or the patient with sepsis presenting with high temperature, low blood pressure and reduced oxygen saturations. Across several disease states, network-based analysis has shown that the loss of healthy variability is a marker of physiological strain. In hypoxic settings alterations in oxygen saturation variability and its relationship with other physiological parameters provides information about an individual’s tolerance and adaptivity. In respiratory conditions such as Chronic Obstructive Pulmonary DiseaseOPD, changes in oxygen-saturation variability can help distinguish stable periods from early exacerbations, offering opportunities for earlier intervention in remote-monitoring settings. In parallel, studies of heart-rate variability (HRV) and heart-rate complexity show similarly promising correlations. These findings illustrate a recurring pattern: disease shifts the body from a flexible, adaptive physiological state to one that is more regular and less responsive, a change that can be captured through continuous-signal analysis and network physiology. Looking ahead, expanding access to continuous monitoring through wearables, both in hospital and at home, creates an opportunity to integrate these insights into everyday clinical practice. Network-physiology metrics could enhance risk stratification, support early warning systems, and help personalise treatment decisions and support precision medicine. “Digital twins” form part of this future in clinical applications. This overview aims to highlight the current impact and uses for network physiology. This will be a jargon-free entry point into the methods, emphasizing practical interpretation, limitations, and realistic pathways for integrating network physiology into everyday care and future research.

## 1 What network physiology entails

Network physiology reframes the human body not as a collection of isolated organs, but as a dynamic network of interacting systems whose moment-to-moment coordination underpins health and disease. Rather than capturing heart rate, respiratory rate, oxygen saturation or blood pressure as static “snapshot” values, it focuses on how the signals from these systems fluctuate over time. Additionally, how the underlying physiology interacts, as reflected by these measured signals, using tools from nonlinear dynamics and network science. Foundational work in this area has shown that different physiological states are characterised by distinct patterns and relationships, supporting the concept of a dynamic “Human Physiolome” (Ivanov, 2021; Bashan et al., 2012; Schöll et al., 2022).

In current clinical practice, most clinicians might observe these fluctuations in a patient’s observations and dismiss them as noise. However, network physiology has demonstrated that variability is not noise. Pattern-analysis of oxygen saturation variability (OSV) in healthy individuals has shown that entropy and related metrics carry information about mean oxygen saturation (SpO_2_) and underlying control mechanisms beyond the average SpO_2_ value itself (Bhogal and Mani, 2017). By measuring multiple systems via oxygen saturation, heart rate, and respiratory rate you can calculate a measure called transfer entropy. Simply put, this measures the impact of a past event on the other variables to clarify if a relationship is random, or if there is measurable information being transferred from variable to variable (Bartsch et al., 2015; Porta et al., 2015; Penzel et al., 2016; Schreiber, 2000).

## 2 Methodology–how can we analyse network physiology

Having established what network physiology aims to capture, this section explains how it does so in a way that is accessible to any clinician.

It is important to note that network physiology encompasses a broad methodological landscape. Approaches for studying interactions among physiological systems include phase synchronisation, coherence, mutual information, Granger causality, and amplitude-amplitude cross-frequency coupling, among other key methodology. These methods differ in their assumptions, data requirements, and clinical applicability. This overview focuses on transfer entropy as one clinically relevant and well-studied example, because it uniquely captures the direction and strength of information flow between signals, making it particularly suited to identifying which physiological system is driving a response (Schreiber, 2000). However, the broader toolkit of network physiology methods should be considered in future clinical translations and reviews.

### 2.1 Step one: observing each signal individually

Physiological variability can be understood from two perspectives. The first is the magnitude of variability; how much a signal fluctuates, which can be measured through conventional statistical measures such as standard deviation or variance. The second is the temporal structure of variability, how fluctuations are organised over time, which is assessed through nonlinear measures including entropy. Entropy does not simply quantify how much a signal varies; rather, it captures the regularity, unpredictability, and complexity of the signal’s pattern. This distinction is important because two signals can have similar overall variability but very different temporal structures, reflecting different underlying physiological states.

Healthy physiology produces complex variable signals (Ivanov, 2021). A healthy person’s SpO_2_ does not sit perfectly still, there is subtle variation in response to breathing, posture, and autonomic activity. Likewise, respiratory rate and heart rate fluctuate from seconds to hours. The first layer of analysis examines each signal on its own. Two properties are measured: variability (how much the signal fluctuates) and entropy (a measure of the regularity and complexity of the signal’s pattern over time) (Ivanov, 2021; Alotaibi et al., 2025). This natural variability is a sign of a system actively regulating itself. When the body is under physiological strain, this complexity tends to fall and the signal becomes more regular and repetitive, even before conventional vital sign thresholds are breached (Ivanov, 2021; Bashan et al., 2012). Clinicians already intuitively understand this. A patient whose SpO_2_ trace is flat and unvarying often prompts concern about correct probe placement or quality. Whilst these individual measurements are helpful, they describe each organ system in isolation.

The benefit of network physiology is what comes next: understanding how the underlying physiological systems interact, as reflected by coordinated changes across these measured signals.

### 2.2 Step two: how the systems communicate–transfer entropy

One of the central analytical tools of network physiology is transfer entropy; a measure of directed statistical dependency between two physiological signals (Schreiber, 2000). For example, does knowing what SpO_2_ has been doing in the last few seconds help predict what the heart rate will do next? If it does, this suggests a directed statistical relationship whereby changes in SpO_2_ are associated with subsequent changes in heart rate though this should be interpreted as evidence of directed information transfer rather than direct physiological causation (Bhogal and Mani, 2017).

This may sound similar to correlation or traditional statistical methods which can tell you that SpO_2_ and heart rate tend to move together (Porta et al., 2015). However, transfer entropy furthers this: it helps reveal if one measured signal is leading another and how strongly. Is the falling SpO_2_ associated with the heart rate rise, or is there some other factor directing both changes (Porta et al., 2015; Schreiber, 2000)?

Transfer entropy is calculated between different signal pairs which may move in either similar or opposite directions, for example,: SpO_2_↔heart rate, SpO_2_↔respiratory rate, heart rate↔respiratory rate (Bhogal and Mani, 2017). The result is a network diagram: a map of the body’s internal communication, where each physiological variable is a node and each interaction is an arrow between them (Porta et al., 2015; Penzel et al., 2016). The strength of each connection is reflected in the weight of the arrow and its direction reflects which variable leads the other. From the above example, it is possible to estimate network indices that allow identification of which node(s) receive or send the highest amount of information from or to other nodes.

In healthy volunteers studied during normobaric hypoxia, transfer entropy analysis revealed that SpO_2_ acts as the central hub of the cardiorespiratory network by exerting the strongest directional influence on both heart rate and respiratory rate (Jiang et al., 2021). A patient’s SpO_2_ reading might be within normal limits at 94%, but the network structure of their cardiorespiratory system may already show the pattern associated with early deterioration (Bashan et al., 2012; Jiang et al., 2021; Porta et al., 2015). Transfer entropy essentially offers a clearer window into physiology that might not be visible from single-value monitoring alone.

An important consideration in transfer entropy analysis is the time lag between signals. The strength and direction of information transfer can vary depending on the delay over which one signal predicts another. Determining the optimal time lag is necessary for accurate quantification of inter-system coupling and remains an active area of methodological development (Schreiber, 2000; Morandotti et al., 2024).

## 3 Current uses and key clinical findings

The analytical tools described above have been applied across several clinical domains. This section examines the current evidence in three areas: chronic respiratory disease, sepsis, and chronic liver disease. For each, we highlight the specific clinical insights obtained through transfer entropy, including the strength and direction of inter-system interactions and changes in network complexity.

### 3.1 Chronic respiratory disease

In chronic respiratory diseases, including Chronic Obstructive Pulmonary Disease (COPD) and asthma, transfer entropy is clinically relevant because these conditions involve dysregulation of interconnected physiological control systems (Alotaibi et al., 2025). Traditional measures such as spirometry and blood oxygen saturation provide separate, isolated measures of clinical condition but fail to capture the dynamic interactions between systems. In contrast, network physiology reframes these illnesses as a failure of coordinated interactions between organ systems, rather than dysfunction of isolated organs themselves. The discovery of this network of systems and the concept of physiological coupling (Bartsch et al., 2012) has given rise to a number of possible clinical applications ranging from early warning systems to risk stratification and prognostication.

Clinically, transfer entropy and related entropy measures have three main applications in chronic respiratory conditions. Firstly, they can enable detection of preclinical instability; alterations in oxygen saturation entropy have been recorded in COPD exacerbations and have the potential to detect exacerbations before traditional methods of diagnosis (Al Rajeh et al., 2021). Secondly, they enable disease stratification; in COPD, current evidence shows that altered entropy patterns correlate with disease severity. For example, reduced entropy in heart rate variability (HRV) and airflow measures reflects impaired autonomic and respiratory control, with progressive reductions observed as disease severity increases (Alotaibi et al., 2025). Thirdly, they provide insight into cardiorespiratory coupling, offering a potential tool to monitor systemic involvement and treatment response. In the context of COPD, entropy related measures derived from SpO_2_ can differentiate and detect an exacerbation, 1 day prior to clinical means (Al Rajeh et al., 2021). Aside from COPD, transfer entropy and other related measures have been used to map the directional flow of information in Obstructive Sleep Apnoea (OSA) (Shah et al., 2026). The significant differences in the directional changes of information seen in this early research, suggest that future work could provide deeper insight into the disease condition and possibly offer novel physiological markers to aid diagnostics or monitoring of patients (Shah et al., 2026).

### 3.2 Sepsis and early warning systems

Loss of autonomic coupling is one of the earliest detectable signs in patients with sepsis. New research into Early Warning Systems (EWS) for patients with sepsis is based on the concept that in healthy physiology, measurable parameters such as heart rate, respiration, and blood pressure show complex, coordinated fluctuations. In contrast, early sepsis causes reduced variability and a breakdown (decoupling) of these interactions, known as “decomplexification” (Fairchild, 2013; Gheorghita et al., 2022). Intuitively clinically this makes sense, during states of poor health the body tends towards predictable patterns (tachycardia in response to a low blood pressure in order to maintain cardiac output). By creating physiological network maps, transfer entropy provides a dynamic measure of how effectively these systems communicate.

Furthermore, in critically ill patients with sepsis, reduced information transfer between physiological systems is associated with worse outcomes (Morandotti et al., 2024). Lower transfer entropy values between physiological signals are linked to an increased risk of early clinical deterioration and higher mortality (Morandotti et al., 2024). Specifically, Morandotti et al. demonstrated that transfer entropy calculated between heart rate, respiratory rate and SpO_2_ was independently associated with clinical deterioration and mortality; with the direction of information flow from SpO_2_ to heart rate being particularly informative. Notably, these network indices were independent of the SOFA score, suggesting that network-based measures capture physiological information not reflected in conventional organ dysfunction scores (Morandotti et al., 2024). Although specific clinical thresholds have not yet been established.

By using pre-existing, inexpensive and simple methods of continuous patient monitoring such as SpO_2_, mean arterial pressure (MAP) and respiratory rate, we can demonstrate physiological decoupling and quantify this with transfer entropy measures, recognising preclinical instability and thus improving patient outcomes with timely treatment.

### 3.3 Chronic liver disease

Network physiology is also clinically relevant in patients with chronic liver disease. Chronic liver disease is a multisystem disorder, where outcomes depend not only on individual organ dysfunction but also on the integrity of inter-organ communication (Schuppan and Afdhal, 2008; Bhogal et al., 2019; Bhogal et al., 2018). Applying a network physiology framework to patients with a multi-system illness such as chronic liver disease requires taking into account clinical variables representing hepatic, renal, coagulation, metabolic, and neurological systems.

Clinically, studies using heart rate variability and complexity measures can demonstrate that patients who display a loss of interconnectivity have a poor prognosis. Patients who are more likely to survive demonstrate highly interconnected physiological networks (Bhogal et al., 2019). In contrast, research shows that patients with severe chronic liver disease who do not survive have marked de-coupling, reflecting a breakdown of coordinated physiological regulation. Bhogal et al. showed that specific HRV indices were independent predictors of mortality in cirrhosis, and proposed that heart rate complexity could serve as a co-morbidity factor in liver transplantation selection, effectively functioning as a marker of network integrity (Bhogal et al., 2019; Bhogal et al., 2018). Transfer entropy applied in this context could further elucidate the direction and strength of inter-organ coupling; for example, identifying whether hepatic deterioration drives cardiovascular dysregulation or vice versa.

Transfer based measures and other forms of network analysis could lead to the development of novel prognostic models in chronic liver disease that go beyond traditional isolated variable-based scores such as MELD (Model for End-Stage Liver Disease, a scoring system using bilirubin, creatinine and INR to predict short-term mortality) or Child-Pugh (a classification using bilirubin, albumin, INR, ascites and encephalopathy to grade liver disease severity) and take into account dynamic interactions evident in severe multi-system disease. Additionally, using transfer entropy to identify patients with highly interconnected physiological networks may improve targeting of patients who are more likely to survive organ transplantation, thus improving the clinical use of such a scarce resource.

In practice, when high-resolution physiological signals are unavailable, physiological network mapping can still be performed using novel approaches such as parenclitic network methods. Importantly, this type of network mapping relies on routinely collected clinical data rather than continuous waveform signals, making it readily applicable in clinical settings. In patients with cirrhosis, parenclitic network analysis of routine laboratory variables has demonstrated prognostic value independent of MELD, with deviations along specific variable pairs such as albumin-bilirubin and albumin-prothrombin time predicting 12-month survival (Zhang et al., 2022). This approach has since been extended to critically ill patients with acute liver failure, where physiological network mapping using routine biomarkers can predict survival and identify distinct network clusters that distinguished survivors from non-survivors (Oyelade et al., 2024). More recently, parenclitic network mapping has also shown promise in predicting therapeutic responses; in a substudy of the ATTIRE trial, network topology at baseline identified subgroups of patients with decompensated cirrhosis who did not benefit from targeted albumin therapy, suggesting that network-based stratification could guide individualised treatment decisions (Oyelade et al., 2023).

Just as scoring systems such as EWS, MELD or Child-Pugh are standard clinical practice, this evolving field of network physiology will be incorporated as routine. The above examples whilst limited, show the benefit of using network physiology and how it can improve on patient care.

## 4 Future research areas and potential uses

### 4.1 Wearable devices at home and in hospital

Expanding access to continuous monitoring through wearable devices will create an opportunity to evolve network physiology research from a largely conceptual framework to a clinical tool used in everyday practice. Wearable devices have the potential to enable remote monitoring of patients at home, with physiological data transferred in real-time to clinicians within the hospital (Webster et al., 2022). This is becoming increasingly relevant as healthcare continues to shift towards telemedicine and online consultations, with remote monitoring enabling proactive and personalised healthcare beyond the traditional hospital environment (Lukas et al., 2020).

In parallel, continuous monitoring of hospitalised patients has demonstrated that it can predict early signs of clinical deterioration and support prevention of unplanned intensive care admissions (Eddahchouri et al., 2022). These two features highlight a clear clinical need and demonstrate the substantial potential benefit of their integration into everyday clinical practice. Wearable devices in particular are expected to play an increasingly important role in enabling preventative and personalised healthcare pathways (Webster et al., 2022; Eddahchouri et al., 2022).

### 4.2 The interaction of organ systems–the “physiolome”

The value of continuous monitoring of physiological data is not primarily about generating more data but instead enabling clinicians to move beyond separate, isolated measurements towards dynamic, network-based assessments of how organ systems interact. As illustrated by Plamen Ch. Ivanov, the central aim of network physiology is to construct an integrated “physiolome” that reflects how physiological systems interact as a coordinated, dynamic network rather than as isolated variables (Ivanov, 2021; Bashan et al., 2012).

The objective is not simply to measure physiological parameters such as respiratory rate, heart rate, oxygen saturation, blood pressure or temperature in isolation. Rather, the aim is to understand how the systems generating these variables adapt and interact in response to physiological stress, disease or therapeutic interventions, as reflected by coordinated changes across measured signals (Ivanov, 2021; Schöll et al., 2022; Porta et al., 2015). Shifting the focus from conventional monitoring of individual vital signs to monitoring of coordinated patterns between organ systems provides greater insight into physiological resilience, instability and recovery (Ivanov, 2021; Bashan et al., 2012; Porta et al., 2015; Santiago-Fuentes et al., 2022).

### 4.3 Enhancing risk stratification and early warning systems

As mentioned above incorporating network physiology into routine clinical practice could enhance existing risk stratification tools, support early warning systems and provide a more detailed understanding of patient trajectories, particularly amongst high-risk or complex disease groups (Ivanov, 2021; Eddahchouri et al., 2022). Rather than relying on individual vital signs to cross fixed thresholds, network physiology can adapt traditional risk stratification models by utilising continuous monitoring to capture subtle fluctuations in physiological metrics which may reveal early signs of physiological stress prior to an overt clinical deterioration (Ivanov, 2021; Schöll et al., 2022).

Research on hypoxaemia and chronic respiratory disease suggests that utilising a network-physiological approach of analysis of oxygen saturation variability could help detect early functional changes and signs of clinical deterioration (Bhogal and Mani, 2017; Costello et al., 2020; Jiang et al., 2021). For example, studies analysing patterns of peripheral oxygen saturation in patients with COPD have shown that patterns in variability can help distinguish between stable states and impending exacerbations, highlighting the potential for earlier detection, avoidance of hospital admission and monitoring of disease progression (Al Rajeh et al., 2021). Similarly, studies have demonstrated that heart rate variability and rhythm complexity are important markers in predicting disease severity and prognosis in cirrhosis (Bhogal et al., 2019; Bhogal et al., 2018). Collectively, these findings demonstrate the potential for network physiology to strengthen early warning systems by identifying early physiological instability before traditional vital-sign thresholds are breached (Ivanov, 2021; Porta et al., 2015; Al Rajeh et al., 2021).

Beyond these clinical applications, network physiology methods have also been applied in experiments settings that are directly relevant to clinical translation. For example, studies have examined how directional cardiorespiratory coupling changes across age groups during different sleep-wake states (Borovkova et al., 2022), how cardiovascular network analysis may inform understanding of autonomic dysfunction in depression (Valenza, 2023), and how physiological networks respond to normobaric hypoxia combined with exercise (Bondi et al., 2026). These experimental applications provide further evidence that network-based measures can capture meaningful physiological changes across a range of conditions and may support the development of clinically applicable monitoring tools.

### 4.4 The concept of the “digital twin”

“Digital twins” form part of this future in clinical applications, whereby real-time data is combined with mechanistic models for prediction modelling and therapy planning in high-risk patient groups (Corral-Acero et al., 2020; Sun et al., 2022). With the increasing power of computing and progress within network physiology, computational models will be able to accurately serve as a prognostic or diagnostic tool (Corral-Acero et al., 2020). Current models in cardiology using “Digital Twins” can assess susceptibility to atrial fibrillation (Corral-Acero et al., 2020). It also has shown that in patients where current evidence may be limited, such cardiac resynchronisation therapy (CRT) in patients with intermediate ECGs, digital twins allowed the verification of a novel method in this substrate of patients. Although currently limited evidence, with a growing understanding of these networks future research will allow precision medicine to be more affordable and accessible.

The relevance of digital twins to network physiology lies in their potential to integrate the kind of multi-system, network-based data that transfer entropy and related methods generate. Rather than modelling organ systems in isolation, a digital twin informed by network physiology could simulate the dynamic interactions between systems. For example, predicting how a deterioration in respiratory function might propagate to cardiovascular instability in a specific patient. This integration could enable clinicians to test therapeutic strategies *in silico* before applying them clinically, representing a meaningful step toward personalized and precision medicine (Corral-Acero et al., 2020; Sun et al., 2022).

## 5 Conclusion

Overall, network physiology analysis methods represent a promising adjunct to conventional assessment, enabling clinicians to move from separate, isolated measurements toward dynamic, network-based evaluation of how organ systems interact in health and disease. This has the potential to enable identification of pre-clinical instability, improve delivery of timely treatment, help develop more accurate prognostic models, and measure the response to therapy more accurately.

This overview aims to provide clinicians with a jargon-free entry point into the rapidly evolving field of network-physiology and its potential clinical applications. However, this overview is not a formal systematic review, and as the field expands and more literature is added, this field will benefit from a more focussed systematic review to summarise and highlight key findings.
